# The Israeli Trauma system during wartime - policy and management

**DOI:** 10.1186/s13584-024-00623-x

**Published:** 2024-07-22

**Authors:** Dorit Nitzan, Joseph Mendlovic, Nachman Ash

**Affiliations:** 1 Masters Program in Emergency Medicine, School of Public Health, Ben Gurion University of the Negev, Beer Sheva, Israel; 2 Ministry of Health, Jerusalem, Israel; 3 Department of Pediatrics, Shaare Zedek Medical Center, Hadassah University School of Medicine, Jerusalem, Israel; 4 Department of Health System Management, School of Health Sciences, Ariel University, Ariel, Israel; 5 Israel National Institute for Health Policy Research, Yehud-Monosson, Israel

**Keywords:** Mass Casualty Incident (MCI), Trauma system, Wars, Health system

## Abstract

On October 7, 2023, Hamas terrorists attacked people in their homes, fields, and at a music festival in Israeli communities near the border with Gaza. More than 1,145 men, women, and children were killed, about 1,800 wounded were evacuated to hospitals in the country, and 253 infants, children, women, elderly, and men were abducted. This mass casualty incident (MCI) was the start of a war that is still ongoing. The Israeli medical system, which faced an overwhelming first 24 h, continues to take care of casualties, including those who are injured by missiles that target Israeli residential areas.

Israel has a well-established trauma system, and as a result of the experience gained in this war, the system merited review. This was the topic of a meeting of leaders of the Israeli healthcare system, and it forms the basis of this report. The meeting and report provide a platform for presenting the trauma system management during the war, highlighting the strengths of the system as well as its challenges and lessons learned. The participants also brainstormed and discussed possibilities for future improvements.

## Introduction

On the 7th of October 2023, on a Jewish Holiday and Shabbat, Hamas terrorists from Gaza invaded Israel. They attacked civilians in their homes and at a music festival, as well as soldiers defending the border. It resulted in the deaths of 1,145 people and approximately 1,800 injuries, with close to 500 requiring hospitalization. Additionally, 253 infants, children, women, men, and elderly were abducted into Gaza.

The Israeli Defense Force (IDF), Israeli Ministry of Health (MOH), the Israeli National Rescue Team, Magen David Adom (MDA), and several Israeli hospitals led and provided life-saving operations for the casualties. The IDF, MDA, and citizens carried out the primary distribution (PD) of hospital casualties. At the same time, secondary distribution (SD) to other hospitals was done mainly by ambulance service providers. The hospitals closest to Gaza faced the challenge of receiving many casualties over a short time while functioning under missile attacks and conditions of extreme uncertainty.

This mass casualty incident (MCI) was the start of a war that is still ongoing. The Israeli medical system, which faced an overwhelming first 24 h, continues up to the present to take care of casualties who are evacuated from the field and of those who are injured by missiles that target Israeli residential areas. The healthcare system is facing new challenges, such as providing access to healthcare services for hundreds of thousands of internally displaced citizens. Additionally, the mental health and rehabilitation services are overwhelmed.

These considerations led the Israel National Institute for Health Policy Research (NIHP) to organize a series of meetings to discuss special healthcare issues that emerged during the war. The first was dedicated to the internally displaced people; the second discussed mental health-related challenges that emerged during the war; and the third, the basis for this report, dealt with the policy and management of the Israeli trauma system during wartime.

The meeting featured presentations by leading experts actively managing the Israeli health system, focusing on the trauma system. Following the presentations, a panel discussion was held. The Deputy Director General of the Ministry of Health chaired the debate on developing peripheral trauma services in hospitals from both local and national aspects. Two invited responders opened the last part of the meeting, followed by an open discussion.

To ensure preparedness for future emergencies, there is a need to focus on improving trauma system policies in Israel based on evidence from experience, expertise, and research. This article summarizes the approaches, ideas, and discussions presented at the meeting. It delves into only some of the issues raised.

## Setting the stage

The Director General (DG) of the MOH emphasized the importance of collaboration among all stakeholders, including first responders, hospitals, and defense forces, to enhance Israel’s trauma system. The events of October 7th and the subsequent war highlighted the strengths and weaknesses of the trauma system, as well as the challenges faced by mental health services. He raised the question of whether the outcome of the casualties during the war would have been different if Barzilay Medical Center (BMC) had been a Supra-Regional Trauma Center or if there had been an additional hospital in Beer Sheva in addition to Soroka Medical Center (SMC). The DG mentioned that he is determined to strengthen access to quality healthcare services for those residing in rural and peripheral areas.

## Meeting’s presentations: the Trauma system – past and present

The Head of the General Medicine Division in the MOH reviewed the resources for managing care and hospitalization of war-related casualties. During emergency response, the whole Israeli health system is required to provide quality health care to the injured while responding to the urgent health needs of the general population. She mentioned that one of the Israeli system’s strengths is that public hospitals are considered a national resource managed centrally by the MOH through its Health Emergency Operation Center (EOC).

The Israeli hospitals’ trauma system is organized around three levels of care: (1) Supra-Regional Tertiary Trauma Centers, (2) Regional trauma centers, and (3) Local trauma centers [1]. The MOH determines the trauma service level according to criteria that include the capabilities of the hospital and its experience. In the event of a war or an MCI, hospitals are trained to follow specific standard operating procedures (SOPs) without delay. These procedures include calling in staff to arrive at the hospital promptly, establishing a chain of command, organizing critical services, discharging patients to free up beds, and coordinating the SD of casualties to other hospitals.

The Israeli Home Front Command, a part of the IDF, manages the PD by helicopters. The policy for the PD of casualties during mass casualty events considers the possibility of an attack from both the north and south borders of Israel. Casualties who are taken by vehicles are expected to be brought to nearby hospitals. In contrast, those transported by helicopters are usually taken to more distant trauma centers in the center of Israel unless the patient is in a critical condition. Super-regional trauma centers are prioritized when distributing casualties from the field (e.g., PD). This PD is based on the vicinity of the super-regional trauma center, the injured health status, the hospital situation, the Hospital Load Index, other events taking place, prioritization in spreading the burden among hospitals, and other limitations.

The decision to activate SD from hospitals is based on various factors, including the need to prepare for more casualties. When the hospital is under an extreme load, it may be reconfigured as a triage hospital. All ambulance services nationwide are regarded as a national resource managed by the Ministry of Health’s EOC for SD.

The MOH is responsible for determining the destination hospitals during the PD of casualties. These decisions are based on real-time assessments of various hospitals’ current capacity and load, focusing on the Supra-Regional Trauma Centers. Once the MOH has determined the destination hospitals, the IDF Home Front Command coordinates with the Northern, Southern, and Central Military Commands to assign and manage transfers of casualties to these hospitals.

The EOC is also prepared to deploy additional personnel to hospitals that require assistance. In addition, the MOH’s International Division is also responsible for requesting and receiving aid from countries abroad.

The next presenter was the former director of general surgery at Rambam Medical Center, who provided a historical perspective on the care of the wounded in wars - lessons and insights from a bloody history. Throughout the history of combat medicine, the aims have been to save lives, reduce morbidity and disability, and return soldiers in good condition to the battlefield. Case Fatality Rate (CFR), as well as Killed in Action (KIA) and Died of Wounds (DOW), are both used as indicators for the severity of injuries and the quality of care. Another indicator used is the “waiting time for medical assistance” and its impact on the death rate from trauma. Since Napoleon’s War, the waiting time for treatment in the field has become significantly shorter, with a marked decrease in CFR.

Based on historical experience, experts have distilled the following lessons:

1. Identifying and preventing deaths that could have been avoided, such as managing bleeding, dealing with tension pneumothorax, and addressing airway problems at the tactical level.
2. Placing medical teams and resuscitation devices closer to the battlefield, enabling the rescue of soldiers with complex injuries who would not have survived in previous wars.
3. Understanding the nature of trauma and injuries to improve treatment methods immediately and prepare for future wars.
4. Conducting research, studies, and learning from past wars to improve outcomes in the future. It is also essential for high-level leaders to engage in medical matters during wars.

Civil-military synergy is critical for improving research, learning, capacity development, and readiness. The keys to success in the next war lie in improving training, practice, and involvement of professional bodies in the planning, documentation, and evaluation. It is essential to strengthen data recording collection, and analyses,, and set real-time guidelines to save lives, reduce disabilities, and enable a quick return to the battlefield.

The next was the Chair of the Israeli Society of Emergency Surgery and Trauma, who focused on the well-developed and experienced Israeli trauma system. He noted that the trauma system has successfully improved the outcomes for wounded patients and encouraged further investment in its development. The first Israeli trauma unit was established in 1992, followed by many more units in the public hospitals during the next five years. The Israel Trauma Society established the Israeli National Trauma Registry in 1995. Important achievements include the 1996 dissemination of the SOP for transferring injured people to trauma centers, the 2004 criteria for recognizing trauma centers, and its 2019 update.

Israel has 29 trauma centers that provide medical care to its population of 9.3 million people. These centers are spread over 22,000 square kilometers and include 7 Supra-Regional Trauma Centers, 13 Regional Trauma Centers, and 9 Local Trauma Centers. In comparison, England has 22 major trauma centers to serve a population of 60 million people, while the Netherlands has 11 Level 1 trauma centers to cater to a population of 18 million.

Currently, 24 hospitals report to the Israeli National Trauma Registry, treating 93% of total injuries in Israel. Thirty-eight registrars collect and validate 150 fields for each injured person. The data of the National Trauma Registry undergo constant quality control and strict adherence to the standards for completeness and accuracy of the information by the National Center for Trauma Research.

Between 2000 and 2019, the CFR for critically wounded patients at the Supra-Regional Trauma Centers in Israel was markedly decreased, according to the data shown. The speaker also emphasized the importance of training in Advanced Trauma Life Support (ATLS), surgical care, and other crucial professional education to manage trauma patients, such as Extracorporeal Membrane Oxygenation (ECMO) in trauma.

The speaker concluded that to move forward responsibly, it is essential to maintain the National Trauma Registry and the National Council for Emergency Medicine and Trauma and adhere to the trauma centers’ recognition criteria. It is crucial to dedicate resources to developing trauma management capabilities and education, which includes supporting those who choose traumatology as their profession. This can be achieved by offering fellowships and scholarships, assisting in attending conferences, providing research opportunities, and encouraging physicians who devote their lives to treating the injured with grants.

The last two presentations of the first session focused on frontline hospitals. In the first one, the General Director of the Galilee Medical Center in Naharia (GMC) emphasized the importance of recognizing the excellence of the Israeli health system in the current period. He emphasized the importance of examining our current actions and preparations, including the investments made into the system.

The GMC, located near the frontline, has operated out of its underground backup facilities since October 7th, 2023. The hospital operates at half capacity (It uses 414 out of its 775 beds) to ensure it is prepared to handle MCIs. The hospital has cared up to now for over 380 wounded soldiers. About 500 hospital staff members, out of a total of 3,260, have been internally displaced from their homes due to the escalation in the north.

“We are very close to achieving the criteria to be recognized as Supra-Regional Trauma Center status,” said the hospital DG. He emphasized that the process could be completed by adding a heart and thoracic surgery department. The hospital doubled its shock room capacity during the war thanks to the MOH’s and donors’ support. This change required infrastructural improvements and adaptation of the health workforce. He underscored the fact that the hospital is not in “war mode” despite challenges and unbearable events with wounded and dead. He concluded that the current approach to assessing the capabilities of hospitals is outdated and underestimated. Additionally, he noted that communication between different entities involved in emergency medical services is not always timely. Furthermore, the Supreme Hospitalization Authority is ineffective in commanding and controlling the situation. He recommended that although patients with complex injuries should be treated at a Supra-Regional Trauma Center, most of those injured in the north can be treated locally based on the experience of GMC.

The last speaker in this session was the Head of Trauma Services at BMC, focused on evacuation sites for combat casualties. He underscored that according to international professional literature, severely injured patients who receive care at the highest level of trauma center have a 25% reduction in CFR (Case Fatality Rate) compared to those who receive care at non-trauma centers. Most studies showed a better survival rate for trauma patients when treated in high-volume than in lower-volume hospitals. However, the ideal threshold cannot be determined. However, it was shown that rural trauma hospitals could have outcomes comparable to urban hospitals with low patient volume. The use of set of protocols for trauma, early activation of a trauma team, and standardized orders are key to the provision of quality care [2]. A study conducted in India revealed that bypassing the nearest hospital for trauma care could significantly improve survival outcomes for severely injured and time-sensitive cases. However, in some situations, transferring the patient to a different hospital may not be feasible, and the patient must be treated at the nearest hospital [3].

He recommended that transferring patients to more distant medical centers only be done when the expertise required is unavailable at the nearer hospitals or for those with complex injuries. He asserted that each hospital has its definition of complex injuries, which makes it unclear who has the authority to decide on SD of casualties.

## Developing frontline Trauma resources – a panel discussion

The expert panel discussed whether frontline hospitals should receive support to become Supra-Regional Trauma Centers. Opponents of this idea argued that peripheral hospitals might lack the necessary expertise due to low patient volume during routine times. Additionally, Supra-Regional Centers may have less experience and expertise since they would not receive the same volume of trauma patients. They emphasized that the country’s already low number of trauma experts would have to be spread over more sites, leaving some uncovered. They stated that the MOH declared its intention without evaluating its overall systemic impact and did not seek input from national trauma experts, who could have suggested alternative solutions, such as providing support from the current Supra Regional Trauma Centers to the hospitals at the frontline.

Other panel experts supported upgrading peripheral hospitals so they can be recognized as Supra-Regional Trauma Centers. They recommended that the process be handled responsibly, using a step-by-step approach. This will help the MOH and hospitals develop a work plan for capacity building, resource allocation, and structural improvements. They emphasized that recognized trauma centers rely on attracting skilled experts, which will have a positive impact on overall access to quality healthcare services in the country’s periphery. The recent war and the shift in the security reality have highlighted the urgent need to strengthen front-line hospitals, as they might need to operate alone, potentially as “isolated islands”. They agreed with the opponents that cooperation and collaboration between the Supra-Regional Trauma Centers and the frontline and other hospitals is necessary. A gradual and responsible process, closely supervised, can enable a short-term leap forward and later meet the requirements for recognition in the trauma center.

## Looking ahead

During the discussions, it was emphasized that Israel’s frontline hospitals need to improve their preparedness and readiness to address mass casualty incidents and trauma challenges. A concrete work plan with timelines and indicators is recommended, including harnessing the current Supra-Regional Trauma Centers to support the frontline hospitals. The input of the professional organizations is essential, along with the advice provided to the MOH DG by the Trauma National Council.

Using the ongoing emergency as an opportunity, we can enhance trauma services for patients in routine and crisis situations. The NIHP can provide a platform for evidence-based discussions to guide and monitor the interdisciplinary process transparently.

## Data Availability

Not relevant for this paper.

## Abbreviations

BMC: Barzilay Medical Center (The nearest hospital to the northern Gaza Strip located about 15 km north of Gaza is a Local Trauma Center).
CFR: Case Fatality Rate
DG: Director General
DOW: Died of Wounds
ECMO: Extracorporeal Membrane Oxygenation
EOC: Emergency Operation Center
GMC: Galilee Medical Center in Naharia (Located in northern Israel, a few km from the border with Lebanon. It is a Regional Trauma Center experienced in previous conflicts, including the treatment of Syrian casualties during the civilian war in Syria.
IDF: Israeli Defense Force
KIA: Killed in Action
MCI: Mass Casualty Incident
MDA: Israeli National Rescue Team, Magen David Adom
MOH: Israeli Ministry of Health
NIHP: The Israel National Institute for Health Policy Research
PD: Primary Distribution
SD: Secondary Distribution
SMC: Soroka Medical Center (A Supra-Regional Trauma Center in Beer Sheva, which is the nearest hospital to the middle and southern parts of the Gaza Strip, located about 50 Km east of Gaza)
SOPs: Specific Standard Operating Procedures
